# Information Security Risk Assessment in Hospitals

**DOI:** 10.2174/1874431101711010037

**Published:** 2017-09-14

**Authors:** Haleh Ayatollahi, Ghazal Shagerdi

**Affiliations:** Department of Health Information Management, School of Health Management and Information Sciences, Iran University of Medical Sciences, Tehran, Iran

**Keywords:** Hospital Information System, Computer security, Risk, Risk assessment, Information system, Medical informatics

## Abstract

**Background:**

To date, many efforts have been made to classify information security threats, especially in the healthcare area. However, there are still many unknown risks which may threat the security of health information and their resources especially in the hospitals.

**Objective:**

The aim of this study was to assess the risks threatening information security in the hospitals located in one of the northwest cities of Iran.

**Method:**

This study was completed in 2014. The participants were information technology managers who worked in the hospitals (n=27). The research instrument was a questionnaire composed of a number of open and closed questions. The content validity of the questionnaire was confirmed, and the reliability of the closed questions was measured by using the test-retest method (r =0.78).

**Results:**

The results showed that among the information security risks, fire found to be a high probability/high impact risk factor. Human and physical/environmental threats were among the low probability risk factors. Regarding the information security safeguards used in the hospitals, the results showed that the use of the technical safeguards was the most frequent one (n =22, 91.7%) compared to the administrative (n =21, 87.5%) and the physical safeguards (n =16, 66.7%).

**Conclusion:**

The high probability risk factors require quick corrective actions to be taken. Therefore, the underlying causes of such threats should be identified and controlled before experiencing adverse effects. It is also important to note that information security in health care systems needs to be considered at a macro level with respect to the national interests and policies.

## 1 INTRODUCTION

Currently, the vast majority of organizations are exposed to a variety of internal and external security threats, such as the manipulation and theft of critical information. Other security threats might be related to the natural disasters and unintentional mistakes of computer users which may lead to devastating consequences [1]. In 2014, Insurance Information Institute in the United States of America reported that 783 data breaches hit business (33.3%) and medical/healthcare organizations (42.5%) [2]. In 2013, Cisco reported that 99% of Android devices were targeted by mobile malware and 71% of Android users encountered with all forms of web-delivered malware [3]. In another report about cyber security trends and challenges, it was revealed that in 2014, 64% of organizations indicated that their security infrastructure was up to date and constantly upgraded. However, in 2015, that number reduced to 59% [4]. This evidence shows that organizations are facing a greater attack surface, the growing proliferation and sophistication of attack models, and more complexity within the network [3].

Similar to other organizations, healthcare organizations are at risk of information security threats. Meanwhile, they are encouraged to use and share electronic health information. They are especially vulnerable targets for data breaches due to the value of health information. Therefore, protecting health information seems to be more challenging than before in the healthcare organizations [5].

Generally, health information security deals with three aspects; namely, protecting patients' data confidentiality, ensuring data integrity, as well as assuring data availability. Ignoring any of these aspects may cause a number of problems, such as legal issues or financial losses for hospitals and health care providers [6-8]. By contrast, improving information security will increase the confidence of patients and clinicians, and may lead to the better use of the health data [6, 7, 9].

Although many efforts have been made to classify information security threats, especially in the healthcare area, there are still many unknown risks which may threat the security of health information and their resources [10]. The most common threats to the information security are unauthorized use of software and computers for communications and illegal activities. The discharged employees can be another threat to data integrity and to overcome this issue, the users' access level should be controlled. In addition, the data integrity can be threatened by hackers, unauthorized users and Trojan horses [7]. Therefore, it is important to identify the information security risks in hospitals to be able to cope with the potential damages in the future. In fact, to minimize losses caused by a variety of security threats, information security risk management is necessary [1]. The purpose of information security risk management is to protect the security in the systems which store, process, or transfer organizational information [11]. In order to manage the risks, there should be a plan to assess the severity of threats and to determine the potential risks [7]. In fact, the process of risk assessment or risk analysis is the first step in the process of risk management [11-13].

There are several methods for assessing information security risks and most of them include identifying threats and vulnerabilities, analyzing the probability and impact associated with the known threats, and ultimately, prioritizing the risks to determine the appropriate level of training and controls necessary for effective mitigation [14]. For example, the IT-GrundsChutz method, which was proposed by the Federal Office for Information Security in Germany, classified the threats to five groups (force majeure, organizational shortcomings, human error, technical failure and deliberate acts). In this method, safeguard measures were infrastructure, organization, personnel, software and hardware, communication and contingency planning [15]. The NIST SP 800-30 is another method, in which the recommendations of the National Institute of Standards and Technology have been considered as a guideline for a comprehensive risk assessment program. In this method, the process of risk assessment is the first phase of the process of security risk management and includes nine steps: 1) system characterization, 2) threat identification, 3) vulnerability identification, 4) control analysis, 5) likelihood determination, 6) impact analysis, 7) risk determination, 8) control recommendations, and 9) results documentation [15, 7]

In Iran, although a number of studies have been conducted about the information security in hospitals [16, 17]; few studies have focused on assessing health information security risk factors and underlying causes of them. This paper aimed to use the NIST SP 800-30 guideline to investigate information security risks in the hospitals. The findings of this study can be used to improve the performance of information technology department and health information security in the hospitals.

## 2 METHODS

This was a mixed methods study which was completed in 2014. The participants were the managers of the information technology departments of the hospitals located in one of the cities in the north-west of Iran (n =27). However, three hospitals were excluded from the study due to the lack of cooperation and finally, 24 IT managers participated in the study. Due to the limited number of participants, no sampling method was used. In order to collect data, a questionnaire was designed based on the literature review and the NIST SP 800-30 guideline [7, 18-20]. The questionnaire had three sections, personal information (4 questions), systems' characteristics and information security status in the hospitals (8 questions), and risk identification. The last section included natural disasters (6 items, e.g., fire, earthquake), human threats (12 items, e.g., hackers, terrorisms), and physical/environmental threats (6 items, e.g., network cable disconnection, chemical spill). Each of the participant was asked to determine the likelihood of the threat/risk occurrence on a three-point likert scale (high=1.0, medium=0.5, low=0.1). Similarly, the impact of each threat/risk had to be determined on a three-point likert scale (high=100, medium=50, low=10). The open-ended questions were considered to ask the participants about the underlying causes of each threat, current solutions, and future control solutions. The content and face validity of the questionnaire was confirmed by four experts in the field of health information management and medical informatics. The reliability of the Likert scale questions was examined using the test-retest method (r =0.78). To analyze data, both quantitative and qualitative methods (thematic analysis) were used.

In order to identify the level of risks for information security, three methods have been suggested. These are quantitative, semi-quantitative, and qualitative methods. In the quantitative approach, the numerical value of the risk impact and the risk probability are calculated and the risks are determined. In semi-quantitative assessment, the risks are classified according to their impacts and the likelihood of occurrence. The qualitative methods explain the likelihood of impacts and are used when calculating the numerical value of risks is difficult [12]. In this study, the quantitative approach was used to identify the risks (Table **1**) [8]. As Table **1** shows, the risk scores between > 50 and 100 require a rapid corrective action plan. The risk scores between >10 and 50 needed a corrective action to be taken in a reasonable time. The risks scores between 1 and 10 could be accepted without taking any action [9].

## 3 RESULTS

As noted before, 24 IT managers who worked in 24 hospitals took part in this study. The mean age of the participants was (37.0± 6.2) years old and most of them were men (83.3%, n =20). More than half of the participants (87.5%, n =21) had an educational background in computer science. In terms of the work experience, most of the participants (75%, n =18) had a work experience of 15 years or less. In this section, nine steps of the risk assessment process are summarized.

### 3.1 System Characterization

The results showed that among different information systems used in the hospitals, the use of financial information systems (n =24, 100%) and admission, discharge, transfer (ADT) systems (n =22, 91.7%) had the highest frequency. The most common computers were desktop computers (PC) (n =24, 100%) followed by the laptop (n =13, 54.1%) and in most cases, each information system had more than 20 users (n =23, 95.8%).

### 3.2 Threat Identification

As noted before, the questionnaire used in this study was designed based on the literature review. The questionnaire included three categories of the information security threats in hospitals. These categories were natural disasters (e.g., fire, earthquake, and flood), human threats (e.g., hacking, terrorism, and spy), and physical/environmental threats (e.g., power outage, chemical spill, and inappropriate ventilation).

### 3.3 Vulnerability Identification

The results showed that some of the underlying causes of natural disasters like fire included old electrical wiring, old networks for electric power transmission, and the lack of fire or smoke alarm systems. The underlying causes of human threats included inappropriate platform of networks, a lack of firewall, a lack of proper physical, technical, and administrative safeguards, and a lack of access to a strong and up to date antivirus. Regarding the physical/environmental threats, the related causes could be an inappropriate structure of the networks, careless computer users and other staff, an inappropriate place for computers and related equipment, inadequate ventilation, and making changes and repairs in the buildings without communicating with the department of information technology.

### 3.4 Control Analysis

Regarding the information security safeguards used in the hospitals, the results showed that the use of the technical safeguards was the most frequent one (n =22, 91.7%) compared to the administrative (n =21, 87.5%) and the physical safeguards (n =16, 66.7%). Overall, about half of the hospitals (n =12, 50%) used the physical, technical, and administrative safeguards to protect information security simultaneously. The most common security control methods included the preventive control actions, such as access control and user authentication (n =22, 91.7%) and the detective control tests (n =20, 83.3%).

### 3.5 Likelihood Determination

Among natural disasters, earthquakes (0.47+0.36) and fire (0.41+0.30) had the highest likelihood and flood (0.11+0.08) had the lowest likelihood of occurrence. Among human threats, computer viruses (0.49+0.37) and intentional removal of information (0.3+0.35) had the highest probability of occurrence. In contrast, the extortion and financial abuse (0.1+0) followed by sending rude emails (0.13+0.18) had the lowest likelihood of occurrence. Among physical/environmental threats, the disconnection of network cables (0.46+0.39) and the leakage of fluid from the roof or pipes (0.41+0.33) had the highest probability and chemical spills on the computers (0.13+0.11) had the lowest likelihood of occurrence.

### 3.6 Impact Analysis

Among natural disasters, fire (61.66+41.46) and earthquake (45.83+36.47) were found to have the highest impact on the information security and storm (15.4+19.7) was found to have the lowest impact. Among human threats, the intentional remove of information (48.75 +42.56) and computer viruses (44.16+37.17) were reported to have the highest impact on the information security and sending rude e-mails (10+0) was found to have the lowest impact. Among physical/environmental threats, the network cable disconnection (45.41+43.93) and fluid leakage from the roof or pipes (45+41.8) were found to have the highest impact and chemical spills on the computers (13.3+11.2) was reported to have the lowest impact.

### 3.7 Risk Determination

Among natural threats, the risk of fire was assessed at a high level, and overall, the risk of human and physical/environmental threats was evaluated at a low level (Table **1**).

### 3.8 Control Recommendations

In order to control the risk of fire, the use of early warning fire and smoke detection systems in different areas of the hospitals and power system automation were suggested. Regarding human threats, defining access level, training computer users and applying administrative, technical and physical safeguards were recommended. The results also showed that to reduce the risk of physical/environmental threats, the use of physical safeguards and appropriate ventilation and cooling equipment in the IT rooms is of high importance.

## 4 DISCUSSION

Security is an important issue when dealing with information, particularly in the health care settings where the nature of information is critical and confidential [21]. Although implementing absolute security is impossible, a security plan is necessary to attain an appropriate or a reasonable level of information security in different organizations. In this case, various parties, such as the individuals, private organizations and companies, and the government agencies will be more confident to be involved in information sharing and taking steps towards a digital world [2]. Currently, information systems and computers are the most important assets in each organization that must be protected due to the value of information. Moreover, there is a direct relationship between the complexity of an organization, its interaction with other companies, and the importance of the generated information. As a result, all organizations are required to adopt an information security risk management approach to be able to identify the potential threats and risks to the information security [14].

In the health care organizations, the advances in information and communication technologies (ICT) have caused health information to be confronted with new security and privacy threats [22]. As a result, many healthcare organizations aim to upgrade the security of their information systems to protect their databases against unauthorized access [21-24]. Since it is impossible to control all security threats, the need arises for a systematic documented method to prioritize the risks and provide mitigation plans [25]. Overall, the process of information security risk management supports the organizational strategic objectives and enables the staff to identify the risk factors around the information processing chain [12]. As noted before, the risk analysis is the first step of the process of risk management, and is a structured and systematic effort to identify the risks and their impacts [14].

In the current study, health information security risks were investigated and the findings showed that among natural disasters, the highest probability of occurrence and the highest impact on information security belonged to fire. Generally, Iran is prone to disasters and it is ranked as one of the most disaster prone countries in the world with floods, drought and earthquakes being the most frequent natural disasters [26]. Apart from these, some areas are extremely vulnerable to the possible fire incidents and natural disasters. For example, earthquakes may increase the chance of fire formation. Therefore, fire could be man-made or natural depending on how the fire is started [27] and identifying the preparation priorities and elevating the preparation level of reaction against fire incidents are enormously essential (26). One of the solutions is providing continuous backups of critical data. Backups are integral part of any recovery plan and it is important to make sure that the copies of backups are stored off-site. All of the backups should not be stored in the same location as the servers. If copies of backups are stored in a separate location, there might be an opportunity to restore data, even if a fire completely destroys the building [27].

The findings also showed that among human threats, computer viruses had the highest probability of occurrence. Generally, human threats can be developed in two ways. One way is related to the people who do not follow security guidelines, forget security considerations, and are not aware of the consequences of their work. The other way is related to those who consciously violate the security guidelines to contribute to the occurrence of a risk. (1) According to Jouini et al, viruses and computer worms are threats caused by intentional human actions that can destroy a high level of information and resources [28]. Similarly, Bakhtiyari Shahri and Zuraini suggested that the user’s activities are the biggest threat to the security of information systems [10]. In case of human threats, the employment of dedicated staff and the use of original and updated anti-viruses can be useful. Although the available antivirus software is used to detect and remove the viruses by using various methods, the existing methods are not sufficient as new viruses are created. Therefore, an intelligent threat identification and intrusion detection system is necessary to handle different types of viruses [29].

Regarding the physical/environmental threats, the findings showed that the network cable disconnection had the highest probability of occurrence. Therefore, it is necessary to identify and control the underlying causes of risks to be able to control the consequences. For example, in case of fire, the use of standard server rooms, automatic power outages systems, and fire and smoke alarms can be useful. Moreover, renewing network infrastructure and modernizing cables, continuous monitoring, personnel training, and using high quality equipment are recommended. To improve the physical/environmental safeguards, the use of video surveillance, expert security staff, intrusion detection systems, innovative architectural and engineering approaches are also suggested to avoid external agents and unauthorized staff access to the data centres [30]. Finally, it can be concluded that hospital managers, information technology managers and other policy makers should work together and address the security gaps existing in the hospitals in order to plan properly and to avoid information security challenges in the future.

## 5 LIMITATION

The current study had some limitations. First of all, in this study data were collected from the hospitals located in north-west of Iran. While the results of this research might be only considered relevant to the settings of the study, the transparency of the research method can help other researchers to investigate information security threats in other settings or other countries.

Another limitation might be related to the limited number of the participants. In fact, due to the time and financial constraints, the study was completed in one of the north-west cities of Iran. To ensure the appropriateness of the questionnaire and to be able to compare the probability and impacts of threats, conducting future research with a bigger sample size and in other settings is recommended.

## CONCLUSION

In this study, health information security risk analysis was conducted. Among the information security risks, fire found to be a high probability/high impact risk factor. Human and physical/environmental threats were among the low probability risk factors. The high probability risk factors require quick corrective actions to be taken. Therefore, the underlying causes of such threats should be identified and controlled before experiencing adverse effects. It is important to note that information security in health care systems needs to be considered at a macro level with respect to the national interests and policies.

## Figures and Tables

**Table 1:** Risk-level matrix.

Impact			Risk
High (100)	Moderate (50)	Low (10)	Likelihood
High 1.0×100=100	High 1.0×50=50	Moderate 1.0×10=10	High (1.0)
High 0.5×100=50	Moderate 0.5×50=25	Low 0.5×10=5	Moderate (0.5)
Moderate 0.1×100=10	Low 0.1×50=5	Low 0.1×10=1	Low (0.1)
